# Impact of Genetic and Genomic Testing on the Clinical Management of Patients with Autism Spectrum Disorder

**DOI:** 10.3390/genes13040585

**Published:** 2022-03-25

**Authors:** Christine F. Stafford, Pedro A. Sanchez-Lara

**Affiliations:** 1 Tufts University School of Medicine, Boston, MA 02111, USA; christine.stafford@tufts.edu; 2 Department of Pediatrics, Cedars-Sinai Medical Center, Los Angeles, CA 90048, USA; 3 David Geffen School of Medicine, University of California, Los Angeles, Los Angeles, CA 90095, USA

**Keywords:** autism, autism spectrum disorder (ASD), genetic testing

## Abstract

Research has shown that genetics play a key role in the development of autism spectrum disorder (ASD). ASD has been linked to many genes and is a prominent feature in numerous genetic disorders. A genetic evaluation should be offered to any patient who receives a diagnosis of ASD, including deep phenotyping and genetic testing when clinically indicated. When insurance does not cover genetic testing for ASD patients, the lack of medical utility is often cited as a reason for prior authorization request denial. However, ample evidence exists that genetic testing has the power to change clinical management in many of these patients. Genetic testing that results in a diagnosis guides clinicians to screen for associated medical conditions and can direct targeted medical interventions. Given the potential for clinically actionable results, it is important that genetic testing be available and accessible to all patients with ASD.

## 1. Introduction

Autism spectrum disorder (ASD) is a neurodevelopmental disorder that encompasses a wide range of presentations and severity but is characterized by deficits in social communication and interaction, as well as restricted and/or repetitive behaviors, interests, or activities [1]. Evidence suggests that genetics play a significant role in the development of ASD [2]. Hundreds of genes have been linked to the pathogenesis of ASD, and family studies estimate the heritability of ASD to range from 50 to 90 percent [3,4]. Autism can be a presenting feature that prompts further investigation for certain genetic conditions, and it is estimated that genetic syndromes or chromosomal abnormalities are present in up to 40 percent of individuals with ASD [5].

The American College of Medical Genetics and Genomics (ACMG) recommends that all individuals diagnosed with ASD be offered genetic evaluation and that targeted testing be performed when indicated [2]. When it comes to genetic testing, the ACMG guidelines suggest chromosomal microarray (CMA) testing for all patients with ASD and Fragile X testing in males [2,6]. These ACMG guidelines do recognize that the same approach cannot be applied to all patient cases, and that decisions regarding testing should be tailored to the individual patient [2]. With respect to the utility of the different types of genetic testing for ASD evaluation, Table 1 summarizes the estimated pathogenic yield for CMA, whole exome sequencing (WES), and whole genome sequencing (WGS), respectively, based on previous studies.

Despite the ACMG’s recommendation, insurance does not always cover testing when it is clinically indicated, often citing a lack of evidence showing that, aside from recurrence risks, genetic testing is not medically helpful. A 2021 study that evaluated the outcomes of prior authorization requests for genetic testing showed that, of the denials, over 50 percent were rejected because the test was either not thought to be essential for medical management or was considered to be experimental or investigational [11].

However, when a patient with ASD is diagnosed with a genetic change, there are numerous clinical benefits. In addition to facilitating access to developmental and educational interventions to help manage ASD, a genetic diagnosis can change the clinical management of a patient. A 2020 study that evaluated the pathogenic yield of genetic testing in children with ASD reported that of patients who received a pathogenic result from CMA or fragile X testing, 72 percent received medical recommendations based on those results [6]. These recommendations could be in the form of increased monitoring for the prevention of associated comorbidities, implementing a medical intervention to improve symptoms, or refining treatment options to avoid potentially harmful interventions [12].

In this paper, we review select ASD-associated syndromes to show how diagnosis following genetic testing impacts clinical management of patients and can improve patient outcome. This paper highlights the importance of having genetic testing accessible and available to patients with ASD.

## 2. Screening for Comorbid Conditions

Diagnostic results from genetic testing can help clinicians screen for and anticipate the development of comorbid conditions associated with a specific disorder, helping to target patients for early interventions and promote disease prevention.

Fragile X syndrome (FXS), caused by trinucleotide repeat expansions in the *FMR1* gene in 99 percent of cases, is the most common single-gene cause of ASD [13,14]. Impairment in cognitive function is a prominent feature of FXS and manifests as ASD in up to 67 percent of males with FXS [15,16]. FXS is associated with several medical concerns for which patients should be monitored, including feeding issues secondary to hypotonia, recurrent otitis media, and connective tissue manifestations such as joint laxity, inguinal hernia, and mitral valve prolapse [17,18,19]. In addition, up to 20 percent of patients with FXS have seizures, and males with FXS and ASD are more likely to have seizures than those with FXS alone [20].

Rett syndrome, an almost exclusively female genetic disorder, typically manifests as regression in developmental milestones, such as loss of speech [21]. Confirmation of the diagnosis is performed via genetic testing that shows a mutation in the *MECP2* gene [22]. ASD is prevalent in patients with Rett syndrome, and in fact, patients are often initially diagnosed with idiopathic ASD [23]. Growth failure is a significant concern in Rett syndrome patients, and management focuses on monitoring nutrition and GI health, as well as comprehensive assessment and monitoring of bone health [24,25].

Caused by the absence of a functioning copy of the maternally inherited *UBE3A* gene on chromosome 15, Angelman syndrome is considered a syndromic form of ASD as many patients also meet the diagnostic criteria for ASD [26,27]. Seizures occur in the majority of patients and are often severe [28]. As a result, recommended management for Angelman syndrome includes an EEG after initial diagnosis for screening [29].

ASD is present in 25 to 50 percent of patients with the neurocutaneous disorder Tuberous Sclerosis complex (TSC) [30]. As TSC results in the formation of tumors in multiple organ systems, surveillance is an important part of disease management. A major manifestation of TSC is the development of cortical tumors that can cause epilepsy, and patients should be closely monitored with EEG [31]. Brain imaging to monitor for subependymal giant cell astrocytoma, renal imaging, and baseline echocardiography and electrocardiography to evaluate for rhabdomyoma and arrhythmia are also recommended [31].

## 3. Medical Intervention

Diagnostic genetic testing for ASD can also impact clinical management by prompting the initiation of a medical intervention. While ASD itself may not be curable, syndromic ASD may have associated features that can be treated medically, and treatment may even extend to improvement of ASD symptoms.

GLUT1 deficiency is an ASD-associated disorder that results in impaired glucose transport across the blood–brain barrier, causing epilepsy, movement disorders, and developmental delay in patients [14]. Diagnosis of GLUT1 deficiency is confirmed by identification of a mutation in the *SLC2A1* gene [32]. The seizures that result from GLUT1 deficiency tend to be unresponsive to antiepileptic medications, and patients are instead treated with a ketogenic diet, which has been shown to effectively treat both the seizures and gait disturbance [14,33]. It is recommended that the ketogenic diet be initiated in these patients as early as possible due to the increased cerebral metabolism of the developing brain [32,33]. As a result, a delay in the diagnosis of GLUT1 deficiency can have an adverse impact on long term patient prognosis and outcome [32].

Timothy syndrome is a congenital long QT syndrome caused by a mutation in the *CACNA1C* gene [14]. It is characterized by QT prolongation, syndactyly, and developmental delay, which manifests as ASD in up to 70 percent of cases [1,34]. Disease management includes treatment with medications to maintain a normal QT interval as well as the avoidance of QT prolonging medications [14]. Consequently, a delayed or missed diagnosis in this high-risk patient population may result in sudden death that could have been prevented [34].

In their 2020 paper, Kreiman and Boles describe five case studies of patients with ASD and genetic mutations in whom genetic testing directly impacted patient management and resulted in an improvement of symptoms [3]. Following the identification of the genetic change, each patient was given specific pharmacologic therapy to target the pathway affected by the patient’s specific mutation [3]. In three of the five cases (a patient with a *TRAP1* mutation given granisetron, a patient with a *CHAT* mutation given donepezil, and a patient with a *SLC6A8* mutation given cyclocreatine), significant improvement was noted in the patients’ ASD-related symptoms [3]. In the remaining two cases, patients experienced improvement in their somatic symptoms [3]. A patient with a *GLS2* mutation who received α ketoglutarate had greatly reduced pain and fatigue, and a patient with a *AANAT* mutation who received melatonin saw normalization in his sleep disturbance [3]. These examples serve as compelling cases for genetically testing patients with ASD given the potential for targeted therapy to improve quality of life.

## 4. Concluding Remarks

The above syndromes represent only a select few of the many ASD-associated genetic disorders. See Table 2 for other examples of syndromic ASD with medically actionable diagnoses. Genetic consultation is recommended following ASD diagnosis, with testing recommendations at the discretion of the clinician. Although genetic testing does not guarantee a diagnosis in every patient, a positive result has the potential to influence a patient’s medical management. Whether it be via targeted screening and monitoring for comorbid conditions or through implementing pharmacologic or other medical interventions for symptom management, these result-based recommendations can drastically improve a patient’s quality of life and in some cases can be lifesaving.

## Figures and Tables

**Table 1:** Estimated Pathogenic Yield of Genetic Testing by Sequencing Method.

Sequencing Method	Proportion of Subjects with Pathogenic Result	References
Chromosomal Microarray (CMA)	9–10%	[2,6]
Whole Exome Sequencing (WES)	16–23%	[7,8]
Whole Genome Sequencing (WGS)	11.2–21.1%	[9,10]

**Table 2:** Examples of Syndromic Autism Spectrum Disorder (ASD) with Medically Actionable Diagnoses [2,35].

Angelman syndrome	CHARGE syndrome	Cornelia de Lange syndrome
DiGeorge syndrome	Fragile X syndrome	GLUT1 deficiency syndrome
Neurofibromatosis 1	Prader–Willi syndrome	Rett syndrome
Smith–Lemli–Opitz syndrome	Smith–Magenis syndrome	Sotos syndrome
Timothy syndrome	Tuberous Sclerosis	Williams syndrome

## Data Availability

Not applicable.
